# Safety and efficacy of concomitant holmium laser enucleation of the prostate with transurethral endoscopic management of symptomatic large bladder diverticulum: revisiting a historical technique in the modern era with literature review

**DOI:** 10.1007/s00345-025-05844-6

**Published:** 2025-08-23

**Authors:** Aravindh Rathinam, Ansh Bhatia, Maggie Meyreles, Hasim Bakbak, Johnathan Katz, Robert Marcovich, Hemendra N. Shah

**Affiliations:** 1 Desai Sethi Urology Institute, University of Miami Miller School of Medicine, 1120 NW 14th Street, Miami, FL 33136 USA; 2 Department of interventional radiology, University of Miami Miller School of Medicine, Miami, FL USA

**Keywords:** BPH, HoLEP, Bladder diverticulum, Endoscopic management, Bladder diverticular ablation, Diverticular neck incision, Bladder outlet obstruction

## Abstract

**Introduction:**

Acquired bladder diverticulum (BD) is typically managed using open, laparoscopic, or robotic approaches. Although transurethral techniques demonstrated favorable outcomes in the 1970s and 1980s, they have largely fallen out of favor. This study revisits transurethral endoscopic management of large, symptomatic BD, combined with Holmium laser enucleation of the prostate (HoLEP) for patients with benign prostatic obstruction (BPO) and coexisting BD.

**Methods:**

We retrospectively reviewed patients who underwent combined HoLEP with transurethral endoscopic management of bladder diverticulum (C-HoLEP–TUBD) between May 2017 and January 2025. Following HoLEP, the diverticular neck was circumferentially resected, and the diverticular mucosa was fulgurated using bipolar cautery. Follow-up cystography was obtained 6–12 weeks postoperatively and perioperative data were collected and analyzed.

**Results:**

Six patients (mean age: 72 + 7.46 years) underwent the procedure. The mean prostate volume was 91 ± 43 cc, and mean BD size was 8.57 ± 5.28 cm. Preoperative Qmax averaged 4.76 ml/s, and mean post-void residual (PVR) was 372.8 cc. At 3-month follow-up, Qmax improved to 25.74 ± 22.4 ml/s, and PVR decreased to 22 ± 23.24 cc. Mean diverticulum size reduced to 1.7 ± 1.19 cm, representing an of 81.89 ± 15.09% reduction. No patient had a residual diverticulum > 2 cm or developed related symptoms during follow-up.

**Conclusion:**

C-HoLEP–TUBD is a safe and effective technique for managing large, symptomatic bladder diverticula in patients with BPO. This combined approach offers significant improvements in urinary function and diverticulum size. Further multi-institutional studies are warranted to confirm these findings and evaluate long-term outcomes.

## Introduction

90% of bladder diverticula (BD) occurs in adults, with 70% of cases associated with benign prostatic obstruction (BPO) [1, 2]. It is estimated that 6% of men with bladder outlet obstruction (BOO) have an associated BD [3]. Since most acquired BD are believed to result from BPO, the American Urological Association guidelines for managing lower urinary tract symptoms (LUTS) due to BPO recommend evaluating men with BD for evidence of BOO [4]. Diverticula with large capacity and narrow necks are particularly prone to complications such as urine retention. Notably, a BD diameter greater than 5.15 cm is an independent risk factor for acute urinary retention in patients with benign prostatic hyperplasia (BPH) [6]. However, it is well-established that not all bladder diverticula, even when large, require surgical intervention [3, 7].

Development of transurethral resection of the prostate (TURP) for BPO led to a more conservative approach to BD management [5]. Currently, surgery is typically reserved for symptomatic BD—those causing recurrent urinary tract infections, bladder stones, tumors, or ipsilateral hydronephrosis [2]. Surgical options include open, transurethral, laparoscopic, and robotic-assisted approaches [1]. Endoscopic techniques such as diverticular fulguration or neck resection were first described nearly five decades ago [8, 9]. When performed concurrently with TURP, these procedures offer advantages including minimal blood loss, shorter hospital stays, reduced analgesic requirements, and avoidance of staged surgeries [8, 10, 11]. Despite these benefits, transurethral techniques have largely fallen out of favor with the rise of laparoscopic and robotic alternatives and are now mostly reserved for frail patients who are unfit for longer procedures [1]. Since 1992, only a single case report and one case series of 39 patients over 14 years have reported outcomes of combined TURP with endoscopic BD management [10–12].

Over the past three decades, Holmium laser enucleation of the prostate (HoLEP) has emerged as a widely accepted, size-independent, and hemostatic alternative to TURP for the treatment of benign prostatic obstruction [13, 14]. Although it has previously been combined with laparoscopic extraperitoneal bladder diverticulectomy [15], HoLEP has not yet been studied in conjunction with transurethral endoscopic management of BD. Given the advantages of HoLEP, we present our institutional experience of concomitant HoLEP with transurethral endoscopic management of symptomatic large BD (C-HoLEP -TUBD). To the best of our knowledge, this is the first study to report outcomes from this combined approach. We also provide a comprehensive review of the literature on transurethral BD management. We believe that our findings support a renewed role for the classic transurethral technique in the contemporary management of BD.

## Methods

### Study design and population

This retrospective study included all patients who underwent en-bloc Holmium laser enucleation of the prostate (HoLEP) combined with transurethral management of bladder diverticulum between July 2017 and May 2024 at our tertiary care academic center. Institutional Review Board approval was obtained for this study (Protocol #20180511).

### Preoperative evaluation

All patients presenting with LUTS attributed BPO were evaluated using the International Prostate Symptom Score (IPSS) and uroflowmetry, unless already catheter dependent. Patients with PVR exceeding 300 ml underwent further imaging to assess upper urinary tract status. The presence of a large BD on ultrasound or computed tomography (CT), along with a history of recurrent urinary tract infections (UTIs), bladder stones, or ipsilateral hydroureteronephrosis, prompted further discussion about treatment options for BD. These included open or robotic-assisted bladder diverticulectomy (RABD) versus TUBD. Patients who opted for TUBD were counselled regarding the potential need for an additional RABD or open bladder diverticulectomy at a later date if they did not show significant reduction in size of BD and continued to remain symptomatic from BD. Prostate volume was assessed via transabdominal ultrasound, CT, or MRI within one year of surgery. Baseline laboratory evaluation included serum creatinine and PSA. Men with elevated PSA underwent further evaluation through shared decision-making. Patients with positive urine cultures were treated with pathogen-specific antibiotics prior to surgery.

### Surgical technique

All procedures were performed under general anesthesia using the en-bloc HoLEP technique previously described [16]. A single surgeon performed or supervised all cases, with assistance from urology residents and fellows. Two patients with coexisting bladder stones underwent laser lithotripsy before HoLEP.

Following successful enucleation, an intraoperative cystogram was performed to evaluate diverticular morphology and assess for vesicoureteral reflux. Subsequently, the BD was fulgurated using a continuous flow resectoscope and bipolar cautery. Details of this procedure have been previously described [2, 3]. Circumferential superficial resection of the diverticular neck was then performed to facilitate wide-mouth communication with the bladder. Care was taken to avoid injury to the intramural ureter. The procedure was concluded with insertion of a 22 French three-way Foley catheter.

### Postoperative care

All patients were admitted for overnight monitoring and received low-rate continuous bladder irrigation. Complete blood count and metabolic panel were checked on postoperative day one. Patients were discharged with a Foley catheter and instructed to return for a voiding trial within 2 weeks.

### Follow-up protocol

A CT cystography at 6–8 weeks postoperatively to assess for resolution of the BD. At 3 months, IPSS, uroflowmetry, and PVR were evaluated. PSA was also repeated at this time to determine the postoperative nadir. Any complications noted during follow-up were recorded.

### Data collection and statistical analysis

Collected variables included patient demographics (age, BMI, comorbidities), preoperative factors (prostate volume, PSA, catheter dependency), intraoperative details, and postoperative outcomes (catheter duration, hospital stay, complications). Operative time reflected total time in the operating room. The enucleated tissue volume was determined by pathology evaluation. Functional outcomes were evaluated using IPSS, Qmax, and PVR at 3-month follow-up. Incontinence was defined as any urinary leakage requiring pads or protection. Continuous variables were expressed as means ± standard deviation, and categorical data as frequencies and percentages. We also performed a literature review using the PubMed database to identify all English-language studies reporting on the TUBD.

## Results

During the study period, 7 patients underwent C-HoLEP -TUBD. One patient was incidentally detected to have a bladder tumor during his surgery and was excluded from the postoperative analysis. Demographics, pre-op parameters, surgery duration, intraoperative complications and post operative parameters of remaining patients are details in Table 1. Out of 6 patients in our series, 4 had large sized prostates (> 80 cc) and the remaining 2 with medium sized prostates had multiple medical co-morbidities and were on anticoagulants. One patient had two diverticula of mean size 6.2 cm preoperatively. One patient was catheter dependent due to obstructive uropathy causing bilateral hydroureteronephrosis and acute kidney injury. In the remaining patients, preoperative mean IPSS was 21.7, Qmax was 5.15 ml/s and PVR was 229.8 ml.

**Table 1:** Patient demographic and perioperative data

	Case 1	Case 2	Case 3	Case 4	Case 5	Case 6	Mean ± SD
*Demographic and co-morbidity*							
Age (years)	59	78	70	72	83	70	72 ± 7.46
BMI	27.12	29.03	34.14	31.74	39.53	27.02	31.4 ± 4.41
Co-morbidity	HT, CAD	DM, HT,	CAD, DM, HT	CHF	AF	–	
On anticoagulants	Yes	No	Yes	No	Yes	No	
*Preoperative data*							
H/o retention	Yes	Yes	Yes, IF	Yes	Yes	Yes, on CIC	
Prostate size (CC)	32	130	39	91	147	107	91 ± 43
Indication for surgery	RUTI	RUTI	SBD	Ipsilateral HUN	SBD	RUTI	
Diverticulum size (cm) max dimension	4	8.5	8.1, 4.3	13.5	3	18.6	8.57 ± 5.28
Hydronephrosis	No	No	Bilateral HUN	Left HUN	No	No	
IPSS	29	14	NA	20	21	30	22.8 ± 5.9
Maximum flow (ml/s)	4.5	7.6	NA	6.3	2.2	3.2	3.1 ± 1.75
PVR	245	226	NA	433	61	899	372.8 ± 288.30
*Intraoperative factors*							
Surgery duration (min)	126	211	246	238	258	224	217.16 ± 43.45
Complications	No	No	No	No	No	No	
*Postoperative factors*							
Postoperative hemoglobin drop	2.7	1.6	0.1	1.9	2.4	1.9	1.76 ± 0.82
Hospital stay (in h)	18	18	17	19	17	19	18 ± 0.81
3-months follow up							
Maximum flow (ml/s)	17.2	27	7.3	68.4	NA	8.8	25.74 ± 22.4
PVR (ml)	30	42	0	1	0	59	22 ± 23.24
Diverticulum size (cm)	1.9	NA	2, 0	1	NA	3.6	1.7 ± 1.19
% reduction	57.77	NA	75.53,100	92.59	NA	80.6	81.89 ± 15.09
IPSS	4	6	7	8	NA	8	6.6 ± 1.49
PSA (ng/ml)	2	0.13	0.06	1.02	NA	NA	0.80 ± 0.78
Incontinence (no. of pads per day)	3	0	1	1	NA	**0**	1.25 ± 1.08
Complications	–	–	–	Dysuria		–	

*IF* Indwelling Foley, *HUN* hydroureteronephrosis, *RUTI* recurrent urinary tract infection, *SBD* stone in bladder diverticulum, *HT* hypertension, *CAD* coronary artery disease, *DM* diabetes mellitus, *AF* atrial fibrillation

**Fig. 1:**
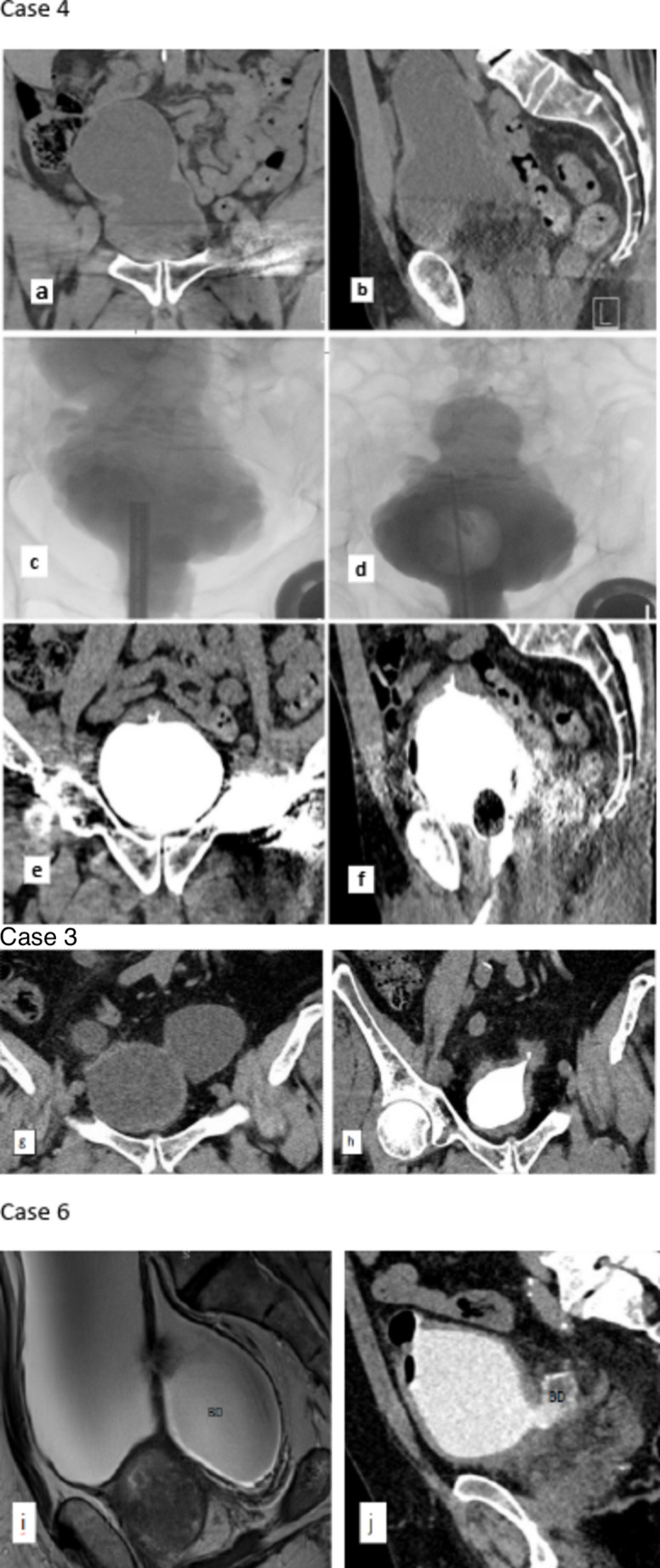
**a**, **b** Preoperative CT scan of case 4 showing BD of size 13.5 × 8.8 cm in coronal and sagittal plane respectively. **c**, **d** Intraoperative cystogram showing immediate reduction in maximal dimension of BD from baseline 13.5 to 7.2 cm immediately after TUBD. **e**, **f** Post-operative CT cystogram at 4 months showing complete obliteration of BD in coronal and sagittal plain respectively. **g**, **h** Preoperative CT pelvis of case 3 showing Mickey Mouse BD of size 8.1 (left) and 4.3 cm (right) and postoperative CT cystogram at 3 months showing complete obliteration of right sided BD and residual small left side BD of 2 cm. **i**,** j** MRI pelvis images of case 6 with (**i**) preoperative bladder diverticulum (BD) size 18.6 cm and 3 months follow up CT pelvis (**j**) showing reduction in diverticulum size to 3.6 cm

The indication of TUBD was recurrent UTI (*n* = 3), ipsilateral hydronephrosis (*n* = 1) and stone in BD (*n* = 2). Two patients also had concurrent Holmium laser lithotripsy of bladder stones. The mean total operative time for combined procedures including laser lithotripsy when necessary was 215.8 min. Histopathological examination of enucleated specimens revealed BPH in all patients. All patients successfully completed voiding trials. One patient did not come for a 3-month follow-up visit, but in a phone call he reported to be “voiding well without any issues”. The remaining patients had satisfactory improvement in voiding parameters at 3 months follow up. Nadir PSA level at 3 months was 0.80 ± 0.78 ng/dl. There were no complications during the 3-month follow-up except for dysuria in one patient. At 3 months, the mean IPSS reduced to 6.6 ± 1.49, Qmax improved to 25.74 ± 22.4 ml/s, and PVR reduced to 22 ± 23.24 cc. Diverticulum size decreased by a mean of 81.89 ± 15.09%. None of the patients had residual diverticula > 2 cm in size at 6–8 weeks postoperatively. Figure 1 shows representative pre and post -operative cystograms demonstrating the effect of TUBD on BD. Table 2 provides details of all published literature on TUBD.

**Table 2:** Literature review on transurethral management of BD

Author	Year	Country	Study type	No. of patients	Associated with	Diverticulum size	Treatment	Energy source	Concomitant surgery	Reduction in BD size	Hospital stay (days)	Complications	Peak flow	Operative time(min)	PVR	Catheter days	Follow-up
Orandi [8]	1975	USA	Observational	17	BPH	NA	Fulguration only	Monopolar	NA	5/17 complete, 6/17 smaller, 3/17 improved	NA	NA	NA	NA	NA	NA	3 months
Clayman et al. [19]	1977	USA	Observational	Transurethral group = 6/11 diverticula alone + 5/11 with TURP) - (open group = 14/30 diverticula alone + 16/30 with open prostatectomy)	BPH and alone	2–14.9 cm	Fulguration and neck incision	Monopolar	TURP in 5, open prostatectomy in 16	TUR: 5/6 complete; open: 8/14 complete, 2/14 smaller	8 (TUR), 19.6 (open)	0 (TUR), 1 reflux + 3 fever (open)	NA	85 (TUR), 126 (open)	NA	3–5	3 months
Posta [9]	1977	Netherlands	Case series	10	BPH + sclerosis internal sphincter	NA	Resection of diverticulum neck	Monopolar	TURP	Curative in 8/10	NA	1 case epididymoorchitis	NA	NA	NA	NA	NA
Adachi et al.	1991	Denmark	case series	9 (8 male, 1 female)	BPH	2/9 Large (13.5 cm and 14 cm)	Endoscopic fulguration + TURP	Monopolar	TURP	84–97% reduction	NA	1 epididiymitis	NA	NA	NA	NA	NA
Yamaguchi et al. [20]	1992	Japan	Case series	31	NA	NA	Bladder neck resection + fulguration	Monopolar	NA	84% (26 pts), 60–90% in 5 pts	NA	NA	NA	NA	NA	NA	24 months
Pham et al. [12]	2016	USA	Case report	1	BPH + Retention	Very large	Endoscopic fulguration + TURP	Monopolar	No	Complete resolution	NA	NA	NA	NA	NA	NA	6 months
Pacella et al. [11]	2018	Italy	Case series	39	BPH	7.5 ± 2.4 cm	Transurethral	Bipolar and Monopolar	8 TUIP, 31 TURP	>80% decrease in 30 pts (76.9%)	5 days	2 post-op fevers	NA	65 ± 21.9 min	NA	7 (5–13.5)	3 months
Pacella et al. [10]	2019	Italy	Comparative	33	BPH	7.5 ± 2.4 cm	Transurethral vs. Laparoscopic	Bipolar and Monopolar	TURP	>80% shrinkage: 15/20 (TUR), 13/13 (Lap)	5 (TUR), 4 (Lap)	2 Grade 3 (1 hematuria, 1 ureter injury)	NA	62.5 (TUR), 185 (Lap)	NA	NA	NA

## Discussion

Our study demonstrates that men with enlarged prostates and symptomatic large BD can be safely and effectively treated with the C-HoLEP–TUBD approach. The procedure was not only well-tolerated but also resulted in a significant reduction in diverticular size, with a mean decrease of 81.89 ± 15.09%. No patients had residual diverticula measuring more than 2 cm on postoperative imaging performed within 3 months. Furthermore, the absence of significant post-void residual (PVR) at 3-month follow-up indicated effective emptying of the residual diverticula.

The first transurethral treatment of bladder diverticula (BD) was described by Hartung and Flocks in 1943, highlighting that 75% of patients improved with treatment of bladder outlet obstruction (BOO) alone [17]. In 1977, Orandi et al. introduced transurethral fulguration of the bladder diverticulum (TUFD), achieving complete resolution in five of 17 patients and significant reduction in others [8]. That same year, Posta proposed resecting the diverticular neck, arguing it functioned like a sphincter, and advocated this method to reduce urinary stasis [9]. Vitale and Woodside later supported Posta’s approach, noting it was safe but cautioned against use in large tumors or near the ureter [18]. In 1984, Clayman et al. combined neck incision with mucosal fulguration in six patients, with near-complete resolution in all cases and minimal morbidity [19]. Yamaguchi et al. refined this in 1992, resecting the neck between the 4 and 8 o’clock positions for diverticula > 1.5 cm. In 26 patients, 84% had complete resolution and the rest had substantial volume reduction, with no residual urine or infections over two years. These developments established transurethral techniques as effective, lower-risk alternatives to open diverticulectomy [20].

After a two-decade hiatus, Pham et al. published a case report, and Pacella et al. presented a case series of 39 patients, reporting outcomes of combined TURP or TUIP with TUBD [10–12]. Pacella et al. from Italy modified Yamaguchi’s technique and instead of resecting the bladder diverticular neck from 4 to 8 o’clock, flattened the entire diverticular neck circumference with a rollerball electrode using plasma vaporization while treating diverticula > 4 cm. The authors considered the procedure successful in 76.9% patients with success defined as > 80% reduction in the size of diverticulum at 3- month follow-up imaging [6]. In the present study we noted a mean 82% decrease in diverticular size at 3 months follow up and none of the diverticula were > 2 cm in maximum dimension.

Over the past 2 decades there has been increasing application of laparoscopic and robotic assisted procedures for the treatment of BD [21, 22]. Some authors consider robotic simple prostatectomy in patients with large BD with the goal of addressing both pathologies concurrently and with the same approach [23]. Others have combined TURP with a laparoscopic or robotic approach [22, 24]. Important considerations of these methods are identifying the diverticular neck and preventing ureteral injury leading to reimplantation of the ureter in some rare cases [25, 26]. In a case series of 28 patient who underwent RABD without any procedure for BPO by 13 experienced urologists the average operating time was 106 min with a mean hospital stay of 2.67 days with 14% of the patients having grade II Clavien-Dindo complications. There was an 11% incidence of ureteral reimplantation in this case series [25]. A recent study RABD with TURP in 4 patients showed median operative time of 212 min with mean hospital stay of 4 days [24]. Our study reported a mean operative time of 217.16 ± 43.4 min and a hospital stay of 18 ± 0.81 h, including anesthesia time implying shorter operative time and hospital stay than laparoscopic or robotic approaches. Additionally, aside from prolonged catheterization, it does not impact recovery or increase complications post-HoLEP. RABD also remains a significantly more expensive option compared to transurethral techniques.

Histopathological evaluations of BD have demonstrated that bladder wall and connective tissue thickness is greatest in diverticula associated with BPO, compared to specimens with isolated BD or BPO [27]. The diverticular neck contains more connective tissue and thickened muscle, whereas the outer portion of the diverticulum comprises a thin mucosal wall with fibrosis and lacks muscularis propria. This structural deficiency results in the absence of contractile function and predisposes to urinary stasis [27]. The heat generated during transurethral cautery likely causes immediate mucosal destruction due to absence of muscle, resulting in visible intraoperative shrinkage of the BD (Fig. 1). Moreover, incision and resection of the diverticular neck facilitates effective drainage of residual diverticula, especially when combined with relief of BOO. We also observed a significant reduction in prostate-specific antigen (PSA) levels three months after HoLEP, with a mean nadir of 0.80 ± 0.9 ng/ml. This confirms complete adenomectomy suggests improved voiding dynamics, which likely enhances emptying of any remaining diverticulum—a phenomenon previously noted in other studies, including that by Agarwal et al. [11].

### Limitations

This study is limited by its retrospective nature and single-center, single-surgeon design with a small cohort. Additionally, it lacks a comparator arm evaluating open, laparoscopic, or robotic diverticulectomy and does not compare outcomes to a staged approach in which HoLEP is performed first, and BD is addressed later if needed. The follow-up period is also relatively short. Despite these limitations, this is the first reported experience demonstrating the safety and efficacy of C-HoLEP–TUBD, a technique that allows concurrent treatment of prostates of any size and associated BD, even in medically complex patients.

## Conclusion

Our study demonstrates that C-HoLEP- TUBD is a safe and effective approach for the concurrent management of BPO and symptomatic large BD. This technique can be applied to patients regardless of prostate size and may be particularly advantageous for those with significant medical comorbidities who are at higher surgical risk. Given its safety profile and functional outcomes, this approach should be considered a first-line treatment option in patients with symptomatic BD and BPO. Future studies are warranted to compare this concurrent strategy with staged management—where BPO is addressed first, followed by BD treatment if necessary—to determine optimal timing and long-term outcomes.

## Data Availability

Data is provided when requested.
